# Fusing Appearance and Spatio-Temporal Models for Person Re-Identification and Tracking

**DOI:** 10.3390/jimaging6050027

**Published:** 2020-05-01

**Authors:** Andrew Tzer-Yeu Chen, Morteza Biglari-Abhari, Kevin I-Kai Wang

**Affiliations:** Embedded Systems Research Group, Department of Electrical, Computer, and Software Engineering, The University of Auckland, Auckland 1010, New Zealand; m.abhari@auckland.ac.nz (M.B.-A.); kevin.wang@auckland.ac.nz (K.I.-K.W.)

**Keywords:** person tracking, re-identification, model fusion, re-ranking, unsupervised learning, Kalman filter

## Abstract

Knowing who is where is a common task for many computer vision applications. Most of the literature focuses on one of two approaches: determining who a detected person is (appearance-based re-identification) and collating positions into a list, or determining the motion of a person (spatio-temporal-based tracking) and assigning identity labels based on tracks formed. This paper presents a model fusion approach, aiming towards combining both sources of information together in order to increase the accuracy of determining identity classes for detected people using re-ranking. First, a Sequential k-Means re-identification approach is presented, followed by a Kalman filter-based spatio-temporal tracking approach. A linear weighting approach is used to fuse the outputs from these models together, with modification of the weights using a decay function and a rule-based system to reflect the strengths and weaknesses of the models under different conditions. Preliminary experimental results with two different person detection algorithms on an indoor person tracking dataset show that fusing the appearance and spatio-temporal models significantly increases the overall accuracy of the classification operation.

## 1. Introduction

In several real-world applications of computer vision, the ability to identify an individual and track how they move is critically important. These applications range from more traditional law enforcement and public security to more modern commercially driven video analytics systems that interpret on consumer behaviour. This is often treated as two separate problems, where assigning an identity label to a detected person is independent of tracking the path and motion of that person.

**Figure 1:**
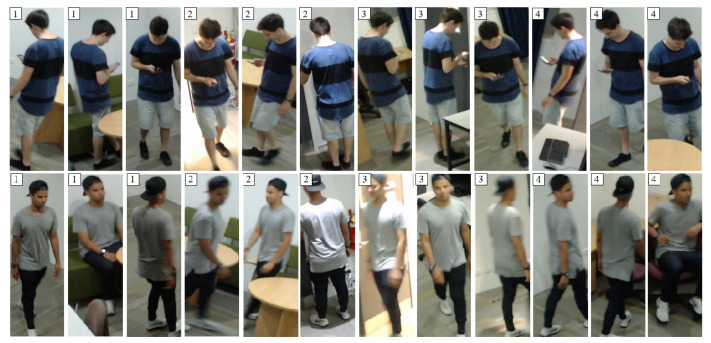
Samples from the UoA-Indoor re-identification dataset extracted with DPM for two classes, with the camera view number shown in the top left.

The idea of using a person’s appearance to discriminate between different identities across multiple camera views is called person re-identification. This is often formulated as a problem of matching images of people as viewed through cameras in different positions and locations, leading to variations in pose, lighting, colour, resolution, motion blur, and obstacles obscuring the person. These challenges are demonstrated in Figure 1, which show how even within the same camera view these factors can cause significant variation in the appearance of a person. However, if person re-identification can be achieved, then the movement of individuals can be tracked by simply forming a list of detected positions over time for each identity class. The problem differs slightly from normal person identification in that the focus is on re-identifying the individual over time to an internal “local” identity label (e.g., person number 6), rather than necessarily matching detected people to a “global” identity (e.g., John Smith).

Alternatively, the notion of tracking the movement of a person over space and time leads to the formation of a spatio-temporal model that can be used to help consistently label the identity of a person. Within the same camera view, it is relatively easy to use a kinematics/physics model to connect person detections between frames of a video and then assign an identity label for contiguous detections. However, there are significant challenges in extending these models between multiple camera views as people move in and out of view; while there has been work in modelling the spatio-temporal relationships between cameras [1], these are not perfect, especially in cases with non-overlapping cameras. Tracking methods also need to separate identities in crowded scenes and deal with significant levels of noise arising from unstable bounding boxes produced by person detection algorithms.

Appearance only uses a subset of the information that is available, and by combining a different type or source of information, the accuracy could be improved. This is particularly relevant if other types of information are strong or reliable where appearance is weak or prone to error. Examples include combining camera information with infrared sensors [2], badge sensors or swipe cards for physical localisation [3], and triangulated wireless Bluetooth signals [4], but these all require additional infrastructure to be installed. A spatio-temporal (position-over-time)-based tracking model presents an alternative type of information that can also be extracted from the camera footage. These two types of information can be used independently to achieve person re-identification or tracking, but could also be used in a complementary way to improve the overall accuracy of the system.

The main contribution in this paper moves towards this goal, using model fusion to combine an appearance model with a spatio-temporal model. The aim is to jointly perform identity classification and tracking, and then fuse the results of both models to achieve higher levels of accuracy. Essentially, both models are designed to produce ranked lists of identities that are most similar to a detected person. These two lists can then be merged together, taking into account the relative strength of confidence in each model, to produce a ranked list of better quality, from which the top result can be taken as the classification output. The presented approaches also have low computational complexity, which helps with real-world implementation (particularly in embedded systems). This is shown as part of a broader modular person tracking system.

In Section 2, we present an approach for fast one-shot/unsupervised re-identification based on appearance, using feature extraction, PCA, metric learning, and Sequential k-Means for classification and model update. In Section 3, a simple tracking system is described, including calibration and mapping, and Kalman Filters for predicting the future positions of people. In Section 4, a comprehensive explanation of how the appearance and spatio-temporal models can be fused together is presented. This includes a re-ranking approach applied using linear weighting, along with a decay function and rule-based system to cover corner cases. In Section 5, experimental results based on the UoA-Indoor dataset are presented, demonstrating that fusing these two sources of information together leads to significantly higher identity classification accuracy. The paper is concluded in Section 6, and avenues for future work are discussed.

## 2. Appearance Based Re-Identification

There are two main approaches for maintaining tracks between cameras—a camera topology-based approach that identifies the sources and sinks of each camera view and tracks moving objects within camera views primarily based on motion and kinematics (or uses auxiliary sensors such as accelerometers to help track the objects between the camera views [5]), and a re-identification approach that matches features between people detected across multiple camera views and tracks primarily based on matching identities [1].

Deep convolutional neural networks are popular for classification [6,7,8], with novel work including joinly searching for people and performing re-identification on raw images with a single CNN [9]. However, deep CNN approaches still suffer from a need for large amounts of training data for each identity, and very computationally expensive, making it challenging to use these approaches in the real-world. Real-time embedded implementations in particular struggle to use CNN methods for practical re-identification. Instead, the more traditional and transparent solution is to extract higher-level features, manipulate or transform that data in some way to make it more separable, and then classify the resultant feature vectors into identity classes representing different people [10,11,12,13]. These approaches are also then more suitable for scenarios with limited or no training data, as one-shot or unsupervised learning methods can be more easily deployed. Scalability is also a key concern, as any system would feasibly have to find separability between hundreds or thousands of identities in a public space.

**Figure 2:**
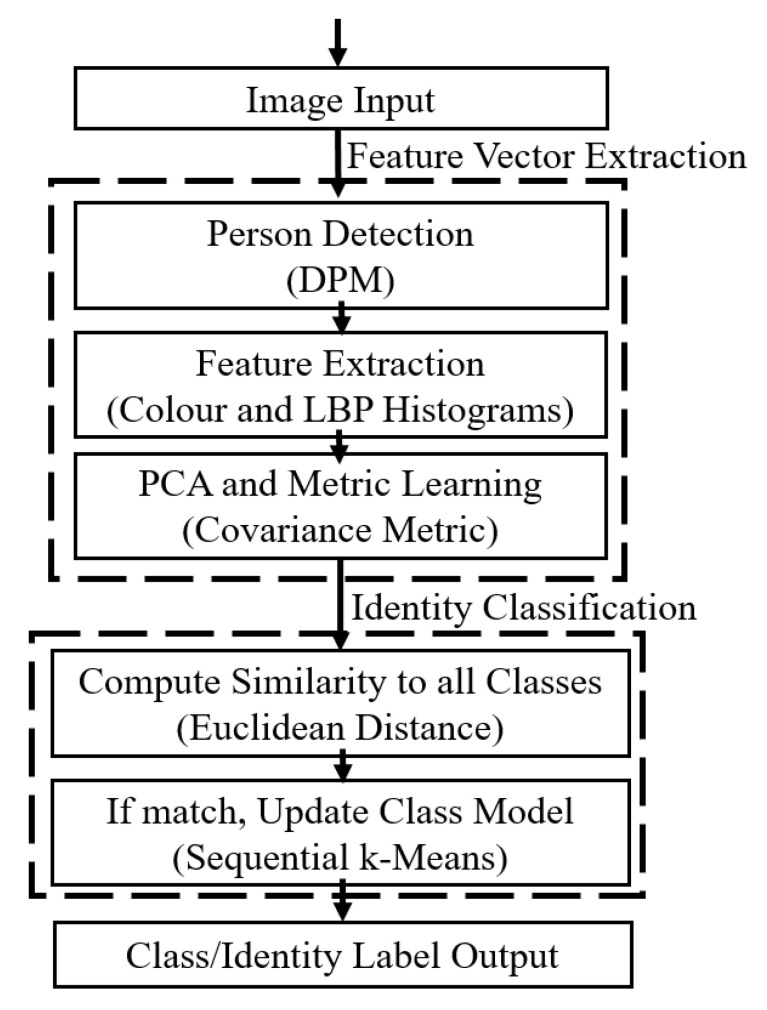
The feature extraction and identity classification processes in the appearance model.

Our fast one-shot re-identification work was covered in our previous publication [14], but is summarised here for completeness. Figure 2 shows the steps taken in this process. Once people are detected within an image, colour (Hue, Saturation, Value (HSV) and texture (Local Binary Pattern (LBP)) histograms that describe the visual appearance of the person are extracted, also known as features [15]. Since we use the Deformable Parts Model (DPM) [16] person detection algorithm, eight parts representing different parts of the body (e.g., arm, leg, torso, head) are available, so the features for each part are extracted separately to provide some contextual information about the features that can be directly compared between person samples.

This results in a long feature vector with tens of thousands of elements. Only some of the information in these feature vectors is discriminatory, i.e., will help to differentiate between one person and another person. For example, some of the elements may relate to textures in the background of every image. With a long feature vector, any set of these feature vectors will also become very sparse, making classification more challenging. Dimensionality reduction can help with this by identifying the most important dimensions/elements and discarding the rest. We do this using Principal Components Analysis (PCA), which takes a set of feature vectors and determines the dimensions with the most variability in an unsupervised way. PCA was shown to be effective in several previous person re-identification applications [10,17,18]. In our previous experiments [14], we show that on our dataset only 32 to 64 dimensions are actually needed for good separability.

Additionally, we then apply a simple metric transformation in order to help make the data more linearly separable. In [14], we show that the simple covariance metric transformation as originally described by Mahalanobis in 1936 [19] actually provides the best result because more sophisticated metric learning approaches tend to overfit to the training data and are less generalisable for previously unseen data. Our experiments show that applying the covariance metric transformation drastically increases the accuracy of an offline SVM from around 18% to 98%. These pre-processing steps result in a short feature vector that is largely linearly separable between the different identity classes.

The feature vector then needs to be classified to produce an identity label. This can be split into two parts—the actual classification step, and the development of appearance models that represent each identity class. In our previous work, person models are formed using a Sequential k-Means approach. Essentially, each identity class is represented by a single cluster mean in the feature space (where there are as many dimensions as there are in the compressed feature vector after PCA and metric learning). The classification step simply calculates the Euclidean distance between the feature vector for a detected person and the cluster mean for each identity class. The Euclidean distance can be used because the original histogram-based features were transformed using the covariance metric transformation into a linearly separable space. The identity class with the lowest distance is therefore the most similar. A ranked list can be produced of all the identity classes and their distances to the detected person. We then update the cluster mean of the top-ranked class with the feature vector from the detected person with linear filtering, such that the new sample contributes to the cluster mean for that identity by a small amount while still allowing past samples to have most of the influence. This helps keep the cluster mean up-to-date as the person moves around and as environmental conditions change, while not allowing noise to have too much of an impact on the model. In experiments on the UoA-Indoor dataset, the Sequential k-Means method outperformed other one-shot or unsupervised learning methods in both accuracy and speed. For more details about the algorithm, please see [14].

## 3. Spatio-Temporal Based Tracking

Within a single-camera view a person’s movements throughout a space can be tracked by estimating the positions of individuals in successive frames, and then connecting points together based on proximity and time to form tracks or “tracklets” (portions of a track). By tracking the person in space and time, a spatio-temporal model of how the person moves is formed. This can be as simple as taking the points in one frame, and connecting each one to the nearest point in the previous frame based on the Euclidean distance. However, the simplicity of this scheme also has many weaknesses; errors can occur if people are walking closely together, or if they walk past each other, or if a person walks behind an object that obstructs that camera’s view of the person momentarily (creating a discontinuity). The most common solution is to add some kinematics physics modelling, essentially estimating the velocity of the person in order to constrain the directionality of the track and bridge discontinuities. Using kinematics is common in tracking moving objects generally, but there are still challenges, such as second-order effects (i.e., acceleration), sudden or abrupt changes in direction, the impacts of noise, and real-time processing requirements [20,21].

**Figure 3:**
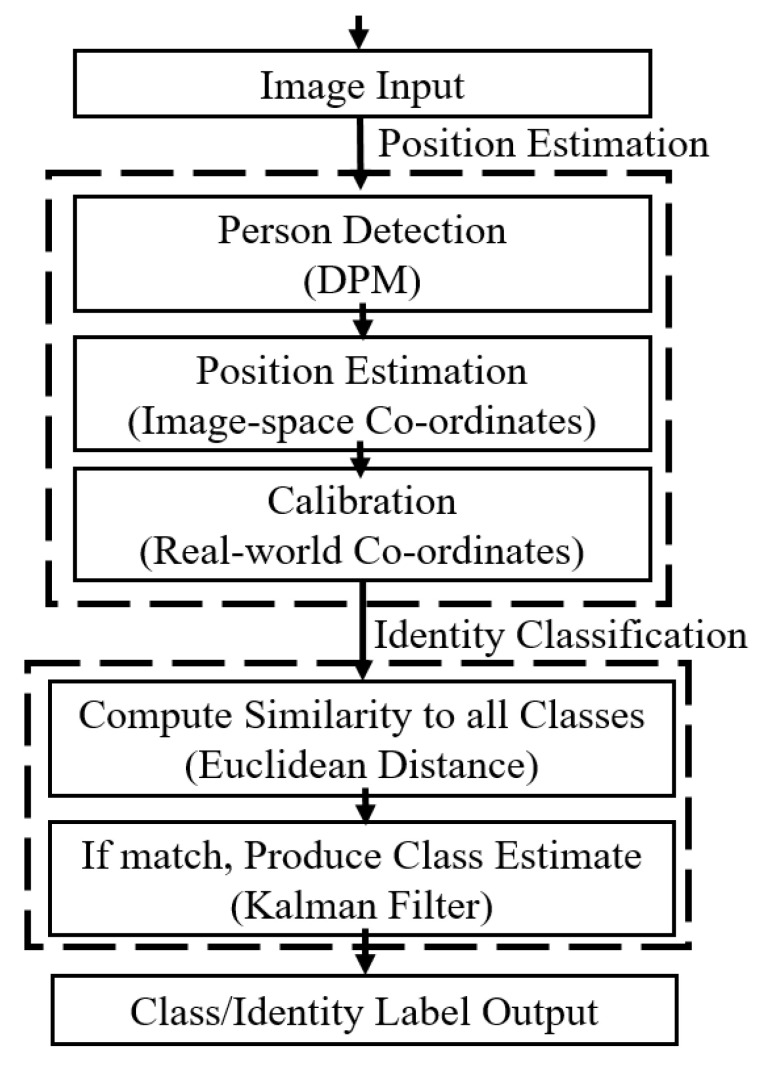
The position estimation and identity classification process in the spatio-temporal model.

The desired output of a tracking system is usually a set of lines or points that represent the path of the object. However, for the purposes of this work, instead of constructing identity-agnostic tracks that just show how people move, the intention is to use person tracking techniques for classification, i.e., giving each track a label corresponding to an identity. This is achieved by using tracking techniques to produce position predictions for each class, and then classifying the input position by finding the closest predicted postion. This is so that the output of the tracking system can be combined with the output of the re-identification system to help determine the identity of the person. Once the position of the person has been estimated in the image/camera view, the spatio-temporal classification is achieved through three main steps shown in Figure 3. These use off-the-shelf algorithms, so they will only be described briefly; readers interested in further details may investigate the references given. First, camera calibration matrices are used to convert the image-space pixel co-ordinate for the person to a real-world co-ordinate on a map [22,23]. Secondly, the position of each person detected in frame N (the current frame) is classified based on their proximity to each of the predictions in frame N-1 (the previous frame). Lastly, Kalman Filters are used to predict the next position of each track in a way that takes the kinematics of the person into account, with robustness against noise [20,24,25].

### 3.1. Calibration and Mapping

Humans are used to seeing maps of physical spaces from a bird’s eye or overhead view (i.e., above the scene, with the field of view towards the floor). However, mounted cameras in ceilings are rarely at that angle—in fact, this would be undesirable in many cases, as the camera would not be able to see the body of the person and would likely only see the top of their head [26,27]. Instead, cameras are often mounted at approximately 45 degrees, halfway between the wall and the ceiling. Calibration is the process of finding a transformation matrix (also known as a projection matrix or homography) that converts the image-world space pixel co-ordinates to real-world space position co-ordinates by using several fixed known points. This can also be described as a process of reconstructing 3D points from the 2D image by determining the camera parameters that transform the real-world space into the image-world space, and then finding the inverse of that transformation. With multiple cameras, this has the added dimension of positioning the camera views relative to each other, usually based on overlaps that allow for topologies to be determined (i.e., where the cameras are, relative to each other). The key to this is assuming a common ground plane (sometimes also called a homography) that is shared between the different camera views. Only the position of the person is needed in this system, so while a 3D-point can be constructed, generally the height is not relevant and an assumption is made that all movement is on a flat ground plane, such that the height co-ordinate can be held constant. This allows for a small number of points with known physical co-ordinates to be used to find a homography between the camera and the ground.

Calibration is a relatively well understood area of computer vision, as it is largely based on single-view geometry and matrix operations, allowing for deterministic operations that are simple enough to be solved by hand. Essentially, the desired output of the calibration operation is a 3 × 3 matrix, which represents the translation and rotation operations that should be applied to each point of the image in order to convert it to the real-world co-ordinate.

**Figure 4:**
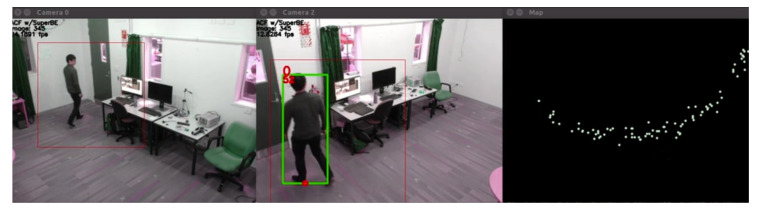
An example of the positions of a detected person being mapped over time. The top edge of the map corresponds to the wall with the door shown in the two camera views.

As people are detected by the cameras, their positions can now be measured using the homographies. First, the position in image-space is measured by taking either the midpoint between the two foot parts in DPM as a reasonable representation of where on the floor the person is currently standing. Then, that pixel point is multiplied by the transformation matrix, which provides a real-world co-ordinate. This co-ordinate can also then be plotted on a map, showing the position of the person as they move throughout the room. An example is shown in Figure 4, where the points are plotted to show the path, which enables full coverage since not all cameras will detect the person or have visibility of the person in every frame. As the map shows, there is significant variation/error/noise—even though a human can easily discern a path that goes through these plotted points, this can present challenges once there are multiple people who might be close to each other, and the wide variation can cause their paths to mix and intersect. This is largely due to the instability of the person detection algorithms; even if a person is standing still, the bounding box is not usually very tight or exact, and has some freedom to move around the person. Since the position is measured based on the bounding boxes, this causes variation in the position measurement, and even a one pixel shift can translate to a shift in the real-world co-ordinate of several centimeters. So while the calibration algorithm itself is relatively consistent and accurate, and produces reasonably good homographies for the shared ground plane between the cameras, the real-world co-ordinates should be considered a noisy and imperfect data source. This can be addressed using a filtering technique that deals with noisy measurements.

### 3.2. Kalman Filters

The use of an algorithm to process the position data in some way has two main purposes: to reduce the level of noise in the position measurements, and to incorporate some kinematics modelling to improve future position estimates. It is important to make a syntactic note here: a measurement refers to the calculated point derived from the sensor (in this case a camera), whereas an estimate refers to the output of further processing that produces a synthetic point that does not exist in the raw data, either from multiple sensor sources or from one sensor source over time. There are several algorithm choices available, but a balance needs to be struck between accuracy and speed. The simplest method is averaging of the measured points from different camera views, but this requires synchronisation of the cameras, and the person is not necessarily detected in all four camera views at any given point in time, introducing the need for more complex co-ordination logic between the cameras. It would also likely fail to model the kinematics of the moving person when the velocity is not exactly constant. Particle filters [28] are another common option, which simulate many possible estimates in the environment (modelled as particles), and iteratively update the probabilities or likelihood of each particle being the true measurement until convergence. However, this is a very computationally expensive method because of its iterative nature depending on the number of particles used, although [29] applied particle filters in a person tracking context and claimed real-time computation of video feeds on a standard desktop PC without parallel processing. Optical flow methods are also common for tracking moving objects, such as the Lucas–Kanade method [30], although this class of algorithm can also be relatively computationally expensive [10]. Perhaps the most popular choice for tracking applications today is the Kalman Filter; it is well understood and used in a wide variety of contexts, from analysing time series signals to navigation and control of robots, vehicles, and aircraft [31,32]. It is a statistical approach using joint probability distributions to combine multiple measurements over time, producing estimates that tend to be more accurate than the original unprocessed measurements. The Kalman Filter is also popular because of its computational efficiency, producing reasonably good results quickly. Kalman Filters were used in person tracking applications in the past [27,33], and seem to be a good candidate for overlapping multi-camera environments where the position measurements from each camera view may disagree and include some error.

The Kalman Filter maintains a model of past information (or state), and combines that with recent measurements to produce output estimates. This is achieved iteratively as new measurements arrive, giving the model more confidence about the true state of the system as more information is provided. The Kalman Filter begins with Gaussian distributions, which model the potential states centered around the first few measurements. As more measurements are introduced, the shape of that distribution is transformed based on the mean and uncertainty of the combined measurements. This is achieved by forming a prediction matrix, and then correcting that prediction with the new measurements to minimise the error between the prediction and the actual measurement(s) using the joint probability distribution, which can be visualised as overlapping Gaussians. OpenCV [34] provides a built-in Kalman Filter implementation, based on the standard formulation given in [24]. In this prototype system, a Kalman Filter is instantiated for each potential identity class, along with an array to store the previously measured points for the purposes of creating tracks. The Kalman Filter is initialised to keep track of four variables: the x position, the y position, the x velocity, and the y velocity (or in other words, two dimensions for position and their first derivatives with respect to time). This can be represented in vector form as [*x*, *y*, $v_{x}$, $v_{y}$]. Since the Kalman Filter used in this spatio-temporal model keeps track of both position and velocity, the probability distribution for the position should be influenced by the velocity estimates as well, although the standard Kalman Filter enforces linear assumptions on these variables.

One issue with this form of the Kalman Filter is that strictly speaking it does not have a notion of time, since it assumes that the measurements are uniformly sampled in time by progressing in logical steps, which may not always be the case in this system. This makes modelling the velocity somewhat challenging, especially since at any given point in time the number of cameras contributing measurements to a particular Kalman Filter is not known a priori. While the effect of this is non-zero, since there are only four cameras in the system, and most of the time only one or two cameras can see a person, the likelihood of multiple cameras contributing points at the same physical time that are being interpreted as being input at successive logical times is relatively low. However, the opposite issue is also challenging to deal with, where there may be indertiminate gaps in time between detections, which can be hard to model using the Kalman Filter because it is difficult to know how many logical steps the Filter should be progressed to compensate for a loss of measurements. Furthermore, this can be somewhat mitigated through good parameter selection. This is because the rate that the distributions change at as new measurements are introduced is governed by several control matrices for the Filter, which can be framed as input parameters for the algorithm, and can reduce the impact of a noisy velocity estimate. While more sophisticated Kalman Filters exist that can better model non-linear movements and more explicitly capture the effect of time, the standard formulation is sufficient for many cases and other forms such as the Extended Kalman Filter introduce significant added complexity and computation time. Incorporating time more explicitly into the model, rather than relying implicitly on the progression of logical steps, would be a key focus for future work on this model.

**Figure 5:**
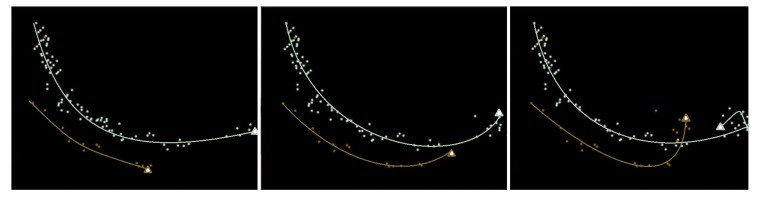
Tracks (drawn by a human) for two people being estimated over time (approx. ten seconds apart). Full circles indicate the measured points from the images, and the triangles at the end of each track indicate the next Kalman Filter estimated position, which is treated as the prediction for that identity class.

The spatio-temporal model for classifying person identities is used in a two-step process: classification and updating. At frame N, when a person is detected, the measurement of the position is compared to all of the predictions from frame N-1, and the person is classified as being part of the class with the most physically proximate prediction based on the Euclidean distance. Then, the measurement at frame N is passed to the relevant Kalman Filter, which produces a new estimate that serves as a prediction to be used in frame N+1. There is a slight caveat in that the Kalman Filter normally needs to “warm up”, because when there are not very many measurements the initial probability distributions can be very large since there has not been enough input data to produce good estimates. This means that for the first 10 detections in each class, the measurements are input to the Kalman Filter, but that measurement is also used as the prediction instead of the estimate from the Kalman Filter. Using the Kalman Filter approach described here, reasonably good predictions can be obtained, as shown in Figure 5, where the tracks and predictions for two people are shown. Person 0 (white) shows a good example of how the Kalman Filter can deal with noisy readings from multiple cameras—after each frame, up to four points could be added (one for each camera view), and the Kalman Filter combines them all (with the past points) to produce a reasonable estimate of the actual position of the person. Person 1 (brown) shows an example of the prediction being very responsive as newer measurements are added, and while the previous measurements have some impact, the more recent points have the largest influence. This spatio-temporal Kalman Filter-based model appears to be reasonably effective for tracking the position of people and helping to classify detected people into their identity classes; evaluation of the accuracy is presented in Section 5.

There are a few weaknesses with this approach to highlight here. The most significant is that the model is dependent on knowing how many people there are in the room, i.e., how many people it should be tracking at any point in time, so that the right number of Kalman Filters can be instantiated. This is achievable in the one-shot learning case where an auxiliary sensor is able to determine when a person has entered and exited the region of interest, but in a purely unsupervised case it becomes very difficult for this model to determine when a new identity class should be created (i.e., a new person has been seen). This can be somewhat alleviated by setting an upper bound during the classification step, where if the closest prediction is still further away than this upper bound, then a new identity class should be created. However, this then exposes a weakness in the entrance/exit areas, where the beginnings and ends of tracks tend to accumulate. Without a way to know how many people have entered or exited the area, it can become easy for a new person entering the space to be assigned a track that belongs to a person who had previously exited the space through the same area, as the prediction from the previous person will be the closest to the current position of the new person. Furthermore, the model faces significant difficulties differentiating between people who are walking closely together—especially because the measurements from the camera images contain significant noise and error. ID switches can become very common, which may not matter while the people are still walking close together, but if their paths diverge then it may be important to ensure that those tracks are assigned to the correct identities from earlier in time. These weaknesses are different to those experienced by the appearance-based re-identification model, so combining these two models together in a novel and complementary way may reduce the negative impacts of these issues.

## 4. Rule-Based Model Fusion

**Figure 6:**
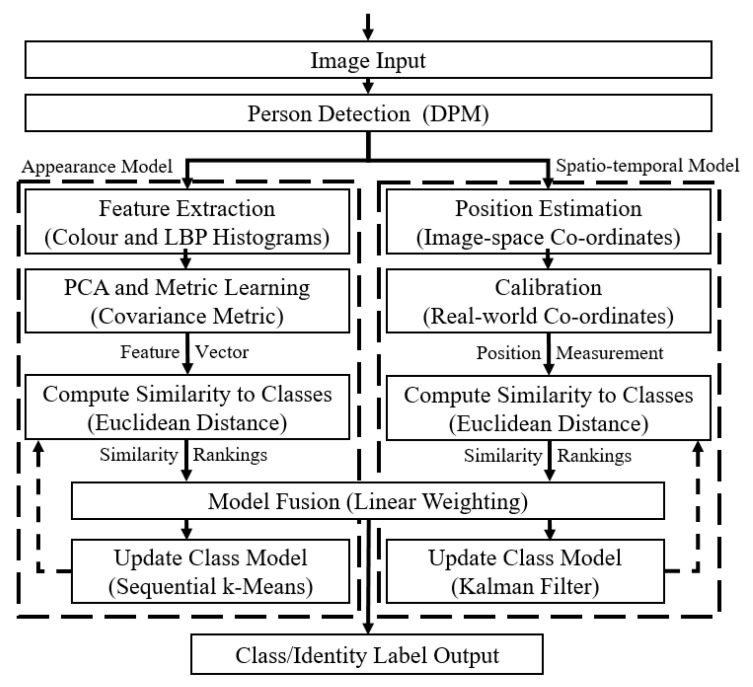
A flowchart of the combined appearance and spatio-temporal models in parallel, leading to the model fusion step, with the class updates feeding back into the classification step for the next detection.

Now that there are two sources of information, they can be combined or “fused” together. Figure 6 shows the architecture that is described in this section, split into the classification and model update steps. Both the appearance and spatio-temporal models are designed to return a ranked list of identity classes, based on the similarity between the probe (newly detected person) and the samples stored in the gallery. The aim of the model fusion module is to combine these two lists, taking into account information such as the relative confidence or likelihood that each class in the list is the correct one, to produce a single output class that is declared the identity number for the detected person. This output is primarily used for subsequent processing such as plotting on a map or keeping records in a log for use in further data analytics. However, importantly, that output class is then also used to update the relevant gallery samples, with separate processes for the appearance and spatio-temporal models, before the next person is detected and classification has to be performed again. It is, therefore, important that the output of the model fusion step is of high quality in terms of accuracy, as erroneous fusion can lead to the models learning incorrectly and increase the likelihood of future misclassification.

Combining person tracking and person re-identification is an idea that holds a lot of promise, yet the literature exploring it is limited. Commonly, spatio-temporal based tracking is used within single camera views, and person re-identification is used to connect tracklets between camera views together. In contrast, the method presented in this paper combines both spatio-temporal tracking and appearance-based re-identification to jointly identify and track individuals as they move throughout a space, making it applicable to both single-view and multi-camera contexts. Previous work has focused on using person re-identification in crowded scenes to help differentiate people within a single view and therefore allow for separate tracks to be assigned to each individual, avoiding ID switch and fragmentation errors. In [35], the PIRMPT system uses colour (RGB), texture (HOG), and shape as appearance features to train a boosted affinity model based on offline training data, and combines this with tracklets to connect them together and produce longer, more stable tracks. A similar idea is used in [36], where they form the task as a Lifted Multicut Problem that combines a probabilistic multicut method to select and connect acceptable tracks from the recorded positions with a supervised CNN-based re-identification model. In these systems, the spatio-temporal tracking usually still takes primacy, with the appearance/re-identification side usually just helping to refine the concatenation of tracklets. The work in [37] presents one of the first systems that treats spatio-temporal tracking and re-identification as two valid sources of information that can be fused together probabilistically, using the Optimal Bayes Filter (based on Bayes Theorem) to combine measures of confidence and dynamically change the level of contribution from each model. In this paper, the weaknesses of both the spatio-temporal and appearance models are explicitly taken into account using a re-ranking process and a rule-based system to determine weights that control the contribution of each model to the final output.

### 4.1. Re-Ranking with Linear Weighting

In the context of person re-identification, the process of re-ranking refers to taking an ordered list of potentially matching identities and then refining that list to increase the likelihood that the correct identity is near the top of the list. For example, POP [38] takes the ordered list of candidates, asks a human to select some definite negative results, and then re-ranks the list by deprioritising similar choices. More recent works have focused on identifying the group of neighbouring/similar classes that should be close together (contextual similarity), which can be used to retrieve classes towards the bottom of the list that should be closer to the top [39,40]. For most person re-identification works, the aim is to produce a good Cumulative Match Curve (CMC), which is based on the correct identity being in the top *r* entries of that list [41]. However, in most real-world applications, having the correct identity in the top *r* entries is not particularly helpful unless r=1; ultimately, the system needs to select one class or identity for labelling so that the appropriate models can be updated. Furthermore, many re-ranking algorithms operate in a supervised manner, using large amounts of data available within a dataset to improve the rankings for that particular dataset; the general applicability of these approaches is questionable, and cannot function in an unsupervised way where training data is not available [40].

The main model fusion task in this paper can be framed as a re-ranking problem where two ordered lists, one from the appearance model and one from the spatio-temporal model are combined to produce one refined list that incorporates the results of both models. There are a family of Ranking Aggregation methods [42], particularly those that relate to optimisation and voting [43], that could be used to achieve this; for example, a Borda Count would allocate points to each position in the list, with the most points allocated to the top (or most likely) classes in each list, and then simply sum the points for each class and re-sort to produce a new ordered list. However, approaches that solely rely on the ordering of the lists suffer from two major problems. First, there is an inherent assumption made that the choices are an equal distance apart from each other (or in other words, the ranking is linear); the third rank is as far away from the second rank as the second rank is away from the first rank. This misrepresents how the list was derived—the classes are ordered based on their distances to the probe (i.e., the person detected in the current frame), and those distances are almost never uniformly distributed. Secondly, since there are only two lists, the likelihood of encountering a Condorcet Paradox is relatively high [44]; the top two options on one list might be classes 1 and 2, and the top two options on the other list might be classes 2 and 1, leading to classes 1 and 2 being equally ranked in the final list with no deterministic/non-random way to separate them.

Two ideas can resolve these two issues. First, the input lists should be accompanied by additional information that gives some sense of confidence about the rankings, for example by including the probe-gallery distances derived for each model, so that the ranking is non-linear. Secondly, not all votes need to be equal, and non-uniform weightings can be introduced to bias the fusion of the two lists towards one or the other, thus avoiding the Condorcet Paradox by ensuring that in the event of a perfect contradiction between the two lists, one list will dominate and win. It makes sense to apply these ideas in this system because the appearance and spatio-temporal models have different weaknesses at different points in time, which should be compensated for by relying more heavily on the other complementary model. Fusing the two lists into one list can be achieved by calculating the similarity score *S* for each class *c* using a linear weighting equation of the form:

(1) $$S_{c}=\beta_{c}ST_{c}+\left(1-\beta_{c}\right)AP_{c}$$

where ST and AP represent some measure of confidence that class *c* is the correct match for the probe identity from the spatio-temporal and appearance models respectively, and β is a dynamic weighting factor between 0 and 1 that biases the similarity score towards one of the two models. The variation in this approach comes from how ST and AP are determined, along with how the weighting factor β changes during system operation. More complicated statistical methods such as using the Central Limit Theorem, Bayes Theorem/Bayesian Networks, and Dempster-Shafer theory [45,46] were also investigated, but they did not seem to yield significantly more accurate results, so they were abandoned since the linear weighting solution was the most computationally efficient due to its simplicity. In this paper, ST and AP are based on the distance between each probe-gallery pair. It should be noted here that smaller distances are better because it indicates that the probe and gallery sample are closer together and therefore more similar, which can be a little counter-intuitive since the resultant “similarity” score is actually a *dis*-similarity score. In principle, the result is a score for each class that incorporates both models, which can then be sorted to produce a new re-ranked list, from which the system selects the top ranked identity as the output class.

A problem is that the ST and AP distances can be at very different scales; for example, since the position difference is measured in mm, the distance is in the order of $10^{3}$, while since the appearance model uses histograms that have already been normalised to be between 0 and 255, the distance is usually in the order of $10^{0}$ to $10^{1}$. This makes sense given that the spatio-temporal and appearance are representing very different things that are not measured on the same scale, so something needs to be done to harmonise the two scales. Therefore, the distances need to be normalised to a common scale based on different scaling factors, otherwise one model will always dominate. It is ideal to normalise the distances to a scale between 0 and 1, but this is challenging because the upper bound on the distances is so large that scaling that to be equal to 1 would force all of the calculated distances to be very small and thus compromise the sensitivity of the model. Instead, the aim can be to set the scaling factors such that they act as approximate thresholds for when the distance for a probe-gallery pair is large enough that it should be considered unlikely to be a match. The effect is that a score of 0 is a perfect match, whereas a score of 1 or larger is very unlikely to be a match. In a purely unsupervised setting, if a probe person has a score of 1 or larger with all of the gallery classes, then it would make sense to create a new identity class, although this is not a perfect measure or condition.

The similarity calculation is also strongly dependent on how β is set. In its simplest form, this can be a parameter set at system initialisation and kept constant throughout system operation. For example, a β of 0.5 would mean that both models contribute equally to the similarity score, while a β of 0.6 would recognise that the spatio-temporal model tends to be more accurate than the appearance model and thus rely on those results more heavily. However, a static weight is too inflexible and fails to properly address the weaknesses of each model. In the general case, the spatio-temporal model is very reliable and produces good classification outputs on its own. However, as soon as more than one person is within the uncertainty range of the position measurements, the spatio-temporal model can easily confuse the identities and switch them or allocate the same identity multiple times within the same frame. In this case, it makes sense to rely on the appearance model more to try and help classify the identities based on their (hopefully) different clothing and general visual appearance. Similarly, if there are multiple people wearing uniforms that make them look visually similar, then the spatio-temporal model could be used to help separate the identities based on their (hopefully) different positions. If a group of people are close together and also have similar appearance, then the system should be able to return an output that indicates it is uncertain, and thus avoid an erroneous classification. To cover the different reasons for why a model may be weak and need more support from the complementary model, β is influenced by two functions: a decay function and a rule-based system.

### 4.2. Decay Function

In the general case, the spatio-temporal model should be relied upon more than the appearance model because kinematic modelling is likely to be more accurate than re-identification within a single camera view, especially as the number of classes becomes larger. However, the most common alternative case is where the person being tracked becomes undetectable for some time, for example by sitting down, or being temporarily obscured by an object, or leaving the camera views and then returning, or because of failures in the background estimation or person detection modules of the pipeline. The longer that a person is not detectable, the more likely that the person has travelled a further distance, and so the more inaccurate their position prediction for the next detection becomes, which is naturally reflected by an increasing level of uncertainty on that prediction. Therefore, it is logical to reduce the reliance on the spatio-temporal model over time if the system has not seen a particular identity for a while. This is achieved by using a decay function, which models an inverse exponential curve for β as time progresses since the last detection. In Equations (1) and (2), β is actually class-specific (*c*), rather than a common weighting that is shared between all classes. This allows for β to be based on ϕ, a variable that keeps track of the time elapsed since the person in the gallery was last detected or seen:

(2) $$\beta_{c}={\left(2\phi_{c}/TF\right)}^{-1}$$

where TF is a timeout factor that is set at system initialisation based on the expected average velocity of the people in the scene. TF is set such that β would be 0.5 when TF seconds have passed since the last time the system saw the person. The expectation is that the system should have seen the person again within TF seconds in order for the spatio-temporal model to be reliable (or more reliable than the appearance model). This variability is only needed because in simulation (or offline processing), while ϕ is based on the physical amount of time that has passed, this does not take into account the processing speed of the system. If stored footage is being processed and the computer is slow or the previous algorithms in the pipeline are complex, then the equation needs to penalise the impact of ϕ appropriately so that β does not decay too quickly relative to the observed speed of the people. On live footage, where the system only samples frames when it has finished processing the previous frame, then the velocity of the person will be the same regardless of how slow the processing speed is. Mathematically, this equation would allow β to be much larger than 1 when ϕ is very small, so it is appropriate to clip β as well:

(3) $$\beta_{c}=\left\{\begin{array}{c} \beta_{max},\mathrm{if}\;\beta_{c}\geq \beta_{max}\\ \beta_{c},\mathrm{if}\;\beta_{max}>\beta_{c}>\beta_{min}\\ \beta_{min},\mathrm{if}\;\beta_{c}\leq \beta_{min} \end{array}\right.\mathrm{where}\;\beta_{max}\geq \beta_{min}$$

where $\beta_{max}$ and $\beta_{min}$ are two weight constraint thresholds that are ostensibly 1 and 0. However, the reason to generalise this equation and allow for other values of $\beta_{max}$ and $\beta_{min}$ is that if they are equal to 1 and 0, then that allows for one of the two models to potentially be completely suppressed. It is unlikely that the confidence in either model would ever be perfect, as there is always the potential for errors to be made. Therefore, slightly more conservative values such as $\beta_{max}=0.8$ and $\beta_{min}=0.15$ may be more appropriate to ensure that both models always have a chance to contribute to the end result. Appropriate values were experimentally determined through hill climbing optimisation techniques, aiming to improve the overall system accuracy; $\beta_{max}$ was between 0.7 and 0.9 and $\beta_{min}$ was between 0.1 and 0.3.

### 4.3. Rule-Based System

While the decay function covers the most common deviation from the general case, there are still many other corner cases that could be covered to help improve the accuracy of the system. Since there are several categorical corner cases, a rule-based system was formulated to further modify β under certain conditions, as shown in Algorithm 1. These corner cases cover initial conditions, dealing with scaling effects, and adding additional constraints when multiple classes are similar to the probe detection. Importantly, the notion of an uncertain result is also introduced, which can be used to block the model update step in order to reduce the likelihood of misclassifications being incorporated into the appearance and spatio-temporal models. This algorithm description also shows that the model fusion approach itself computes in linear time (Θ(n)), with the exception of the ratio matrices which are $\Theta (n^{2})$ but relatively simple to calculate.

#### 4.3.1. Initialising Gallery Models by Camera

At this point, it is important to note that rather than maintaining one global model for each identity across all camera views, a separate model is maintained for each camera view. When a person has been detected for the first time, the model for each camera view is initialised with the same sample, but after that, each camera is allowed to modify its own appearance model for that person. This improves classification accuracy for the appearance-based model, as inter-camera effects such as changes in illumination and viewpoint that contribute to variation between cameras are reduced. This is a reasonably important departure from the standard re-identification problem formulation, which usually aims to re-identify across multiple camera views. However, a robust re-identification approach is still needed, as even within the same camera view there can be significant variation in the person appearance, as previously shown in Figure 1. Since position information and predictions of future positions are also available in this system, the confidence for the appearance model can be reduced if the source camera view has not seen the classified person yet. If the top ranked identity from the spatio-temporal model has not been seen by the current camera, then β is set to $\beta_{max}$ in order to emphasise the spatio-temporal model and rely less on the appearance model (Algorithm 1, Lines 2–3).
**Algorithm 1** Computation and use of β, including the rule-based system to cover corner cases.Set a
β value for each class *c*1: **for** each class *c* in the gallery *C*
**do**2: **if** ethis camera is seeing the ST model’s top ranked identity for the first time **then**3:  $\beta_{c}=\beta_{max}$4: **else if** the scaled appearance distance is very close to zero **then**5:  $\beta_{c}=\beta_{min}$6: **else**7:  $\beta_{c}={\left(2/TF*\phi_{c}\right)}^{-1}$ {the decay function}8:  Clamp $\beta_{c}$ between $\beta_{min}$ and $\beta_{max}$9: **end if**10: **end for**
Cover similarity-based corner cases, overwriting previous β values if necessary11: Calculate $\psi_{AP}$ and $\psi_{ST}$ {the ratio matrices}12: Initialise *w*, $w_{AP}$, and $w_{ST}$ to False {assume good certainty initially}13: **if**
$1.25>\psi_{AP_{1,2}}>0.8$ {the first and second classes appear similar} **then**14: Form a restricted set $R_{AP}$ of classes *c* where $1.25>\psi_{AP_{1,c}}>0.8$15: $w_{AP}$ = True {appearance model has low certainty}16: **for** each class $c\;\epsilon \;R_{AP}$
**do**17:  $\beta_{c}=\beta_{max}$18: **end for**19: **end if**20: **if**$1.25>\psi_{ST_{1,2}}>0.8$ {the first and second classes are proximate to each other} **then**21: Form a restricted set $R_{ST}$ of classes *c* where $1.25>\psi_{ST_{1,c}}>0.8$22: $w_{ST}$ = True {spatio-temporal model has low certainty}23: **for** each class $c\;\epsilon \;R_{ST}$
**do**24:  $\beta_{c}=\beta_{min}$25: **end for**26: **end if**27: **if**$w_{AP}$ and $w_{ST}$ {both the appearance and spatio-temporal models are uncertain} **then**28: *w* = True {declare that the final output will have low certainty}29: **end if**
Calculate the class *c* with the most similarity to the input person>30: **for** each class cϵC
**do**31: $S_{c}=\beta_{c}ST_{c}+\left(1-\beta_{c}\right)AP_{c}$32: **end for**
33: $R=R_{AP}\cup R_{ST}$ {Combine the two restricted sets together}34: **Return** the highest ranking class *c* where cϵR if R≠∅ else cϵC, with $S_{c}$ to represent the similarity score and *w* to indicate the certainty on the result


#### 4.3.2. Scaling Effects

Another change is rebalancing the distances being produced by the appearance model. Since the appearance of a person is very susceptible to noise, the histograms that comprise the feature vector are not very stable. This means that while the threshold for normalisation may not have to be particularly large, the distance values themselves can fluctuate below this threshold. Empirically, it was found that the distance values for the appearance model tended to be in the top 50–60% of the dynamic range. For example, if the normalisation threshold is set to 10, then the within-class distances tend to be between 4 and 10. The 0–4 range of the distance space is essentially unused and wasted. If the distance between a probe image and the gallery image is 5, then this would be normalised to 0.5, even though to a human the images may appear to be quite visually similar. This can become an issue when fusing the appearance and spatio-temporal models, because since the distances are normalised and directly used as a measure of confidence in the rankings, then when the spatio-temporal model uses the lower end of its dynamic range it can easily but potentially erroneously win over the appearance model stuck at the upper half of its range. This can be somewhat alleviated by subtracting a minimum value from each of the appearance model distances and the threshold, thus improving the precision of the remaining range. For example, a floor could be set at 4, and simply subtracted from each of the distance values and the normalisation threshold so that a distance of 5 becomes a distance of 1, with a normalisation threshold of 6, corresponding to a 0.167 normalised score. While this helps the appearance model compete against the spatio-temporal model, any normalised distance (after rebalancing) that is very close to 0 is also extremely likely to be a match. It would be undesirable for an erroneous spatio-temporal distance measurement to cause that to be misclassified, so in this case it is best to set β to $\beta_{min}$ in order to rely mostly on the appearance model and only allow the spatio-temporal model to override the appearance model rankings in very, very extreme circumstances where the spatio-temporal model is very confident in its classification (Algorithm 1, Lines 4–5).

#### 4.3.3. Similar Similarity

**Figure 7:**
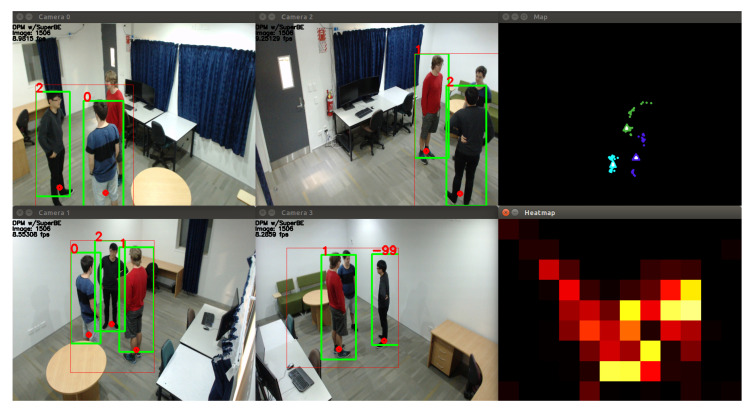
An example of a group of three people being tracked and identified correctly. In each camera view, the thin red box indicates the area that is isolated during background estimation for further processing, the green boxes represent the detected people, and the red circles represent the estimated position for each of those people. They have consistent ID numbers between the cameras (shown in red above each person), where −99 indicates low certainty (and is ignored). The maps show the positions of the individuals, with the top of the map matching the wall with curtains shown in Camera 0 and 2. The top-right map shows the last fifty detections per class as circles and the estimated future position from the Kalman Filters as triangles. The bottom-right heatmap shows the most occupied areas of the room since system initialisation, quantised to 40 cm × 40 cm patches, where lighter/brighter colours indicate higher occupancy.

There are two conditions that cover the most challenging cases for a person tracking and re-identification system; if people look very similar, or are physically proximate, or both, then the chances of the person tracking system producing incorrect labels increases substantially. This is represented numerically as there being multiple classes that produce similar similarity scores between the probe detection and the target model in the gallery. To check for these conditions, this system uses ratios to standarise the comparison of how similar distances are to each other. This is inspired by the use of likelihood ratios in medical sciences for validating diagnostic tests, based on Bayes Theorem [47,48]. Ratios are popular because hard thresholds for determining similarity are inflexible and fail in situations where the scale of the values can change. For example, a 0.1 threshold for declaring two measurements as being similar means very different things if those measurements are in the $10^{-1}$ order of magnitude or the $10^{2}$ order of magnitude. Instead, a ratio (i.e., dividing one measurement by another) reduces those measurements down to a common scale, which can then be compared to a threshold that is applicable across all scales of the original measurements. A ratio matrix ψ can be derived by dividing each probe-gallery distance by each other probe-gallery distance, as shown in this brief example:

(4) $$\left[\begin{array}{ccc} 1 & 2 & 3 \end{array}\right]\to \left[\begin{array}{ccc} 1/1 & 1/2 & 1/3\\ 2/1 & 2/2 & 2/3\\ 3/1 & 3/2 & 3/3 \end{array}\right]\to \left[\begin{array}{ccc} 1 & 0.5 & 0.33\\ 2 & 1 & 0.67\\ 3 & 1.5 & 1 \end{array}\right]$$

where the vector on the left represents three distances, the middle matrix shows how each value in the ratio matrix is derived, and the matrix on the right is the ratio matrix for that vector. The similarity between any two distances can then be determined by how close the ratio is to 1. Since each model produces a ranked list of classes, $\psi_{AP}$ for the appearance model and $\psi_{ST}$ for the spatio-temporal model can be calculated, and then the person tracking system can retrieve the ratio for the first and second ranked classes for each model. If the ratio is close to 1 (approx. 0.8–1.25), then both the first and second rank classes should be considered viable, and sufficiently similar solutions to the classification problem. In fact, there may be more than two classes that are very similar—if the first two classes are similar, then the other ratios for that first ranked class should also be checked. All classes *c* that have distance measurements that are sufficiently similar to the top ranked class can then be placed into a restricted set *R*. For each class *c* in *R*, its β weighting value can then be set to prioritise the appropriate model; if people appear very similar, then prioritise the spatio-temporal model, and if people are physically proximate, then prioritise the appearance model. Importantly, the final re-sorting operation is then limited to only those classes that are in *R*. This logically makes sense; for example, if the spatio-temporal model has identified that the probe image belongs to one of three people standing in a circle talking to each other, then it should use the appearance model scores for those three people only to determine which one it is, rather than allowing another identity to be erroneously selected. The final corner case is where the model fusion module has found both that the first and second rank people are very similar in both appearance and position. In this case, rather than risk misclassification, the output can be declared uncertain (using *w*), so that the person tracking system does not update the gallery models, and can record that as required in any plotting or logging operations. This allows the system to fully resolve the Condorcet Paradox. The logic described in this subsection is presented in Algorithm 1, Lines 11–29 and Algorithm 2, Lines 2–4. Figure 7 shows an example of this rule-based system in action; a group of three people standing closely together are able to be separated based on their appearance, and the system is able to indicate when it is uncertain about the identity of a person.
**Algorithm 2** Model update and class creation with unsupervised learning.1: Receive the Similarity Score $S_{c}$, the class label *c*, and the certainty *w* from model fusion2: **if**
*w* is True {if there is low certainty on the result} **then**
3: Overwrite the class label with −99 to indicate low certainty4: Do not update the models, do not create a new class5: **else if**
$S_{c}>$ New Class Threshold {if most similar class is above the threshold} **then**6: increment the total number of classes by 17: Create a new class ID8: Initialise the spatio-temporal model (Kalman Filter)9: Initialise the appearance model (Sequential k-Means)10: **else**
11: Update the spatio-temporal and appearance models for class *c*12: **end if**


Using a linear weighting approach along with the weight decay function and rule-based system, this model fusion process is able to comprehensively cover a wide variety of scenarios, and take advantage of the strengths of the appearance and spatio-temporal models when appropriate. However, it is important to keep in mind that the required output is a single identity class that matches the identity of the person in the probe image. While the two models may disagree on their rankings, a sophisticated model fusion process is only needed when they disagree on the top ranked class. A speed optimisation to the model fusion system can therefore be made by declaring an output class immediately if both the spatio-temporal and appearance models agree on the top ranked class, skipping the re-ranking process entirely since it is very unlikely that the re-ranking process would produce a different result. This shortcut further reduces the computation time of the model fusion process without compromising the accuracy. The output class can then be used to update the appropriate class in the spatio-temporal and appearance models, using the Kalman Filter and Linear Weighting (as part of Sequential k-Means) respectively.

### 4.4. One-Shot vs. Unsupervised Learning

As far as the model fusion approach itself is concerned, there is no learning as the parameters are not being adjusted during runtime. However, the resultant similarity scores *S* from the fusion module can have a large impact on the learning parts of the system. This is because the main difference between one-shot and unsupervised learning is class creation; in one-shot learning, when to create a new class is given to the system (e.g., when an RFID access tag is triggered to indicate that a new person has entered the scene), whereas in unsupervised learning, the system has to determine itself when to create the new class. In this implementation, when using unsupervised learning, a new class is created if the best similarity score between the probe and the gallery classes is above a set threshold (Algorithm 2, Lines 5–9). This is not particularly flexible, and is strongly dependent on the consistency of the model fusion algorithm, but is probably the simplest way to determine when new classes should be created. Algorithm 2 shows how the system deals with both the model update (which is essentially the learning part of the system) and class creation when using unsupervised learning, following on from the results returned by model fusion in Algorithm 1. Any errors encountered by the one-shot learning approach should only be where the same identity class is given out to multiple people, whereas the unsupervised learning approach would have to contend with that problem as well as the potential of creating extra identity classes and fragmenting a single person across multiple identities. Therefore, it should be natural to expect that unsupervised learning should lead to a lower accuracy rate than one-shot learning, but in some applications unsupervised learning may be the only option available to system designers.

## 5. Experimental Results

### 5.1. Baseline Experiments

**Figure 8:**
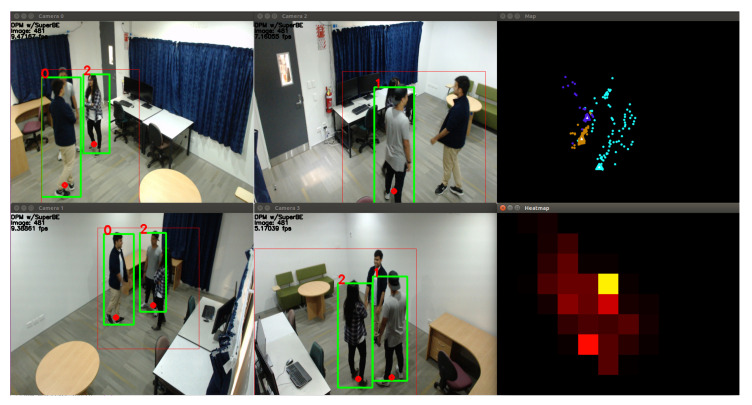
A group of people being tracked, with an erroneous classification due to the people standing closely together and the model fusion algorithm failing to separate the individuals. This figure follows the same annotations and mapping structure as Figure 7.

To test the performance of the overall system, experiments were conducted on the UoA-Indoor dataset [49] on *Walk* (where there is only one person in the room at a time) and *Group* sequences (up to four people in the room at the same time, interacting with each other). The UoA-Indoor dataset consists of footage from four overlapping camera views (as shown in Figure 7 and Figure 8), emulating an indoor office environment. The dataset has footage at a resolution of 1920 × 1080 at 15 frames per second collected from consumer grade webcams. The dataset contains almost three hours of footage, with 19 different identities annotated across 150,000 frames. The 19 identities represent an upper limit on scalability in this experiment, and a larger dataset would be needed to test if these methods work with larger numbers of identities. While people may not be in the room at the same time, the system remembers the identities of people it has seen before. All subjects gave their informed consent for inclusion before they participated in the dataset. Ethics approval was given by the University of Auckland Human Participants Ethics Committee (Ref. 018182, approved 18 November 2016). Experiments were performed on a desktop computer running Ubuntu 16.04 with an i7-6700 CPU with four 3.40 GHz cores and 16 GB of RAM.

Two sets of experiments were run; one with one-shot learning, where the presence of an auxiliary sensor such as an RFID tag indicating when people are entering the space for the first time is assumed, allowing the system to know when to initialise a new class with a single labelled sample, and the other with unsupervised learning, where the system has to determine when to initialise a new class based on similarity score thresholds. There are several parameter values that need to be determined, including thresholds for the appearance and spatio-temporal models for normalisation, score thresholds for when to create a new identity class, and ceiling/floor weights for the β weight values in the model fusion module. These experiments were repeated thousands of times in a parameter sweep, trying uniformly random combinations of the different parameter values in the first pass to try and get a general understanding of the parameter space, and then optimising towards higher accuracy levels in a second pass. Accuracy is measured as the number of detections that are correctly classified; missed detections (false negatives) are ignored for the purposes of this metric. The best accuracy results are shown in Table 1, which clearly show that fusing the spatio-temporal and appearance models together improves the accuracy of the video analytics system overall. The unsupervised learning results are only about 4% points lower than the one-shot learning approach, so taking other engineering requirements into account such as the cost of installing an auxiliary sensor or difficulties in synchronising sensor signals may lead to the conclusion that the unsupervised learning approach is acceptable or even superior for certain applications.

It is important to understand the failure modes in this system to account for the accuracy rate. Analysing the results from the unsupervised learning approach has shown that it does tend to create more identity classes than the true number; in the best results, there are 37 classes in the gallery, even though there are only 19 identities in the dataset. In the accuracy measurements, the system is penalised for these extra identity classes because they are all essentially counted as errors or misclassifications. These extra classes tend to be created when a person appears to “teleport”, i.e., suddenly move very far from where they currently are. This mainly happens due to the person detection algorithm not providing a stable position as the bounding box shifts, and if the box shifts then this can lead to a large change in the estimated position. This should be treated as noise by the spatio-temporal model, so this suggests that the spatio-temporal model is still not robust enough to deal with all types of noise, and could be improved by using a more sophisticated tracking approach since the spatio-temporal classification and model update are not the speed bottleneck in this prototype. This issue does not exist in the one-shot learning approach, since the number of classes is controlled and only increases as each new person enters the camera views. However, for both types of learning, there appear to still be some difficulty in separating appearances when they are very similar, for example, when there are two identities wearing black shirts and black pants. The most significant issue is probably that the spatio-temporal model gets confused by the conglomeration of identities at the entrance/exit areas of the camera views. Even though the model fusion algorithm allows for an output to be uncertain if there are multiple gallery appearances and positions that are similar to the probe sample, this is problematic if a new identity is similar but it has not yet been initialised in the gallery, especially while the Kalman Filter is still warming up. In this case, the video analytics system is likely to misclassify the new identity as one of the existing gallery classes, reducing the accuracy of the system. It should also be noted that only a subset of the relevant parameter space was explored due to time and resource constraints, yet the parameters can have a critical impact on the performance of the system. For example, with model fusion and unsupervised learning, the best result was 63.5% but the worst result obtained was only 3.6%. With further experimentation, better parameters could be found, but this can be challenging as the parameter space appears to be multi-modal (i.e., there are many local minima and maxima), and it is likely that these would still need to be modified for each new operating environment.

Comparing these accuracies to the state-of-the-art results is very difficult; the proposed video analytics system has only been tested on relatively small numbers of people and on a dataset that has not been tested with other methods. If the accuracy results are directly compared, then this system may not appear to be that impressive. However, there are some key strengths that make this a promising avenue for further exploration. The vast majority of modern person tracking systems use supervised methods to artificially achieve high accuracy rates that could never be achieved outside of the laboratory. Even when transfer learning is used (i.e., training on a similar context and then performing fine-tuning training within the real environment), significant amounts of training data from the real environment are still needed, which is often simply not practically available. In addition to this, the computation time for this video analytics pipeline is almost real-time; with DPM, this system runs at about 10 frames per second on a standard PC without GPU acceleration (which includes person detection), which is significantly faster than any modern deep learning or CNN-based system. This speed has to be taken into account when interpreting these accuracy results; systems that can achieve a similar level of speed tend to have much lower accuracy rates, such as [10], which has a re-identification rate of approximately 20–40% for rank-1 (which is only shown in CMC curves; their results tables start at rank-5) with between 57 and 94 identities, and [50], which reports a rank-1 accuracy of 18.7% on their clean dataset with no false positives or occlusions across 462 bounding boxes/person detections.

Only qualitative results are available for the *Group* sequences since there is no labelled ground truth available yet. The screenshot in Figure 8 shows an example of the unsupervised video analytics pipeline using the DPM person detector on one of these sequences, with a different group of people in a different position to Figure 7. While the detection and tracking looks reasonably good, there is one misclassification in Camera 1 where the person in the grey shirt should be person 1, not person 2. Looking at the map on the top-right, it can be seen that the position estimates and predictions are very close together, making classification difficult for the model fusion algorithm.

### 5.2. Comparing Person Detection Algorithms

The video analytics pipeline operates at just under 10fps using DPM when there is a person being detected in the camera views. To demonstrate that our approach is not specific to a particular person detection algorithm or feature extraction scheme, we ran the system with a different person detection algorithm that does not have parts to see how it might impact accuracy. Replacing DPM with the Aggregate Channel Features (ACF) model moves to the faster end of the accuracy-speed trade-off. The ACF implementation used in this research is based on the work of [51,52], with modification of the code to allow for non-maximum suppression and extraction of patches.

A few changes to the pipeline are needed when switching person detection from DPM to ACF. Most significantly, parts-based sampling is no longer available, as extra computation would be required to segment the detected person into semantically meaningful body parts, which would defeat the purpose of using a faster person detection algorithm. Instead, patch-based sampling is used, with 32 patches (four columns and eight rows, with 50% overlap in each direction) to segment the bounding box. This results in a much longer feature vector, but PCA can be used again to establish a transformation matrix that can reduce the feature vectors to 64 elements long, so that it is the same length as the vectors used with DPM. New parameter values had to be found as the appearance features may vary numerically. The same experiments run with DPM are also run with ACF (i.e., random sampling of the parameter space with both one-shot and unsupervised learning). Table 2 shows that the model fusion approach still significantly increases the accuracy over the two models being used separately. Comparative results for unsupervised learning are shown in Table 3; while using ACF is about 7% points lower in accuracy than DPM, it is more than two times faster. Whether the new speed-accuracy trade-off offered by ACF is acceptable will depend on the end application. The difference in computation time between using DPM and ACF also shows how dominant the effect of the person detection algorithm is on the overall computation time. In our analysis of the computation time for each stage of the pipeline, person detection with DPM accounts for approximately 65% of the computation time, while classification and model fusion account for approximately 10%. This is further evidence that the proposed model fusion methods have relatively low computational complexity. The most expensive part of the classification and model fusion part of the system is the appearance classification (feature matching), as it is processing comparatively longer feature vectors than the spatio-temporal classification.

Practically, the accuracy drop between DPM and ACF is caused mostly by there being far fewer detections/observations in ACF; for the unsupervised learning case with model fusion, DPM classified approximately 12,700 detections without high uncertainty, while ACF classified approximately 4800 detections. This is because the ACF detection threshold was set at a relatively high level (60 out of 100), which causes the algorithm to miss some detections (false negatives), but also avoids the worse problem of there being extra detections (false positives). False positives have to be avoided as otherwise they can cause significant changes in the gallery models; the system is robust to the occasional, rare false positive, but as the false positive rate increases the legitimate detections would become swamped. This could potentially be avoided in the future by introducing a separate “false positive class”, as suggested by [50]. When there are fewer detections, the spatio-temporal model tends to suffer more, as the movement of the person appears more discontinuous and more difficult to model using a Kalman Filter. This makes it more likely for the system to erroneously create new classes, leading to more misclassifications and a drop in accuracy. It is also important to note that along with more missed detections, ACF also has less accurate bounding boxes that are less stable (i.e., they are more likely to move frame-to-frame even if the person is standing still) [51], which can also negatively impact the spatio-temporal model.

## 6. Conclusions and Future Work

In this paper, a comprehensive model fusion approach is presented for combining appearance and spatio-temporal information about people for re-identification and person tracking. First, a computationally light person re-identification approach was described. Secondly, a spatio-temporal model was proposed, using calibration and mapping to convert pixel points to real-world co-ordinates, and then using Kalman Filters to help reduce noise and provide position predictions for classification. Thirdly, the model fusion approach was presented, using a linear weighting approach to combine the spatio-temporal and appearance models together to improve the overall accuracy of the system. This was combined with a decay function and rule-based system to help change the weighting on the two models dynamically for different scenarios. The accuracy and computation time were found through experiments on the UoA-Indoor dataset, and show that the model fusion approach significantly improves the accuracy of the system. Lastly, both DPM and ACF person detection algorithms were compared against each other to show that this methodology may have more general applicability.

In terms of future work, perhaps the most significant is to further investigate the use of camera topology as contextual information during model fusion. At the moment, each detection of a person is processed independently; when a camera detects a person, it is classified without taking into account what the other cameras are seeing at that particular point in time, or even what else is being seen by the same camera. It could be possible to constrain identity classification with additional rules, such as “the same ID cannot be allocated twice in the same frame for the same camera view” or “at least two cameras must agree on a classification”. Some preliminary ideas are explored in [53], where multiple ranked lists of people are collected for people seen in the same frame by the same camera, and then classes are penalised down the list if they are also near the top of other lists since the same identity should not appear at the top of multiple lists. It could also be possible to modify the confidence levels and weightings during the model fusion process based on the characteristics of the camera or the environment of a particular camera view; for example, it may be valid to declare that a camera view is particularly saturated by the presence of external lighting (e.g., sun coming through the windows) that makes colour appearance less reliable, so the appearance model should be penalised and the spatio-temporal model more heavily relied upon. Last of all, more options could be considered and implemented for the different pipeline stages; perhaps a particle filter is a better option for the spatio-temporal tracking model, or a small but deep neural network might be more suitable than Sequential k-Means for the appearance model, to improve the accuracy overall.

## Figures and Tables

**Table 1:** System Accuracy with DPM for Person Detection.

Classification Model	Accuracy (%)	
	One-Shot Learning	Unsupervised Learning
Spatio-temporal Only	33.3	31.7
Appearance Only	51.9	48.8
Both Fused Together	65.7	61.6

**Table 2:** System Accuracy with ACF for Person Detection.

Classification Model	Accuracy (%)	
	One-Shot Learning	Unsupervised Learning
Spatio-temporal Only	34.3	30.6
Appearance Only	53.8	47.1
Both Fused Together	69.4	56.7

**Table 3:** Performance Metrics with Unsupervised Learning for DPM and ACF.

Person Detection Method	System Accuracy (%)	Computation Time (ms)	Computation Time (fps)
DPM	**63.5**	102	9.8
ACF	56.7	44.8	22.3
